# Supplementary material for: Characterization of Maladaptive Processes in Acute, Chronic and Remission Phases of Experimental Colitis in C57BL/6 Mice
Source: Biomedicines. 2022 Aug 5;10(8):1903. doi: 10.3390/biomedicines10081903 (PMC9405850; doi:10.3390/biomedicines10081903)
Supplement: Supplementary file 1 [file biomedicines-10-01903-s001.zip › Supplementary Tables 2 and 3.pdf]

Supplementary Table 2A: Data summary of leukocyte flow cytometry analyses within colon *lamina propria*. The table summarizes relative immune cells frequencies of CD45^{+} leukocytes from main Figure 2B. Only subsets showing significant differences in reference to control groups are shown. ▲: increased frequency, ▼: decreased frequency, ■/■ : reference condition, n.d.: population is not detectable.

Lamina propria leukocyte subset as % of CD45+	day 11	day 56	day 6	day 25	day 46	day 67
	control acute	control chronic	acute	acute remission	chronic	chronic remission
B cells	■		▼			
DN lymphocytes	■	■		▼	▼	
Dendritic cells		■			▼	
Macrophages	■	■	▲			▼
Eosinophils	■		▲			
Monocytes	■		▲			
CD4+ T cells	■			▲		
Neutrophils	n.d.	n.d.	▲	▲	▲	▲

Supplementary Table 2B: Data summary of leukocyte flow cytometry analyses within colon *lamina propria*. The table summarizes absolute cells counts of leukocytes from main Figure 2C. Only subsets showing significant differences in reference to control groups are shown. ▲: increased count, ■: reference condition, n.d.: population is not detectable.

Lamina propria leukocyte subset - absolute cell count	day 11	day 56	day 6	day 25	day 46	day 67
	control acute	control chronic	acute	acute remission	chronic	chronic remission
Eosinophils	■		▲			
Monocytes	■	■	▲		▲	
CD4+ T cells	■	■			▲	▲
CD8+ T cells		■				▲
Neutrophils	n.d.	n.d.	▲	▲	▲	▲

Supplementary Table 3: Data summary of soluble mediators from colon homogenates. The table summarizes data from main Figure 3. Only mediators showing significant differences in reference to control groups are shown. ▲: increased concentration,
■: reference condition, n.d.: population is not detectable.

Soluble mediators in colon homogenates	day 11	day 56	day 6	day 25	day 46	day 67
	control acute	control chronic	acute	acute remission	chronic	chronic remission
IL-6	■	n.d.	▲		▲	▲
TNF-α	■	n.d.	▲		▲	▲
IP-10	■	■	▲		▲	
KC	■	■	▲		▲	
MIG	■	■	▲		▲	
MIP-1α	■	■	▲		▲	
BLC		■			▲	
IFN-γ		■			▲	
IL-1α		■			▲	
MCP-1		■			▲	
RANTES		■			▲	
IL-17a		■			▲	▲
MDC		■			▲	▲
