# Supplementary material for: Characterization of Maladaptive Processes in Acute, Chronic and Remission Phases of Experimental Colitis in C57BL/6 Mice
Source: Biomedicines. 2022 Aug 5;10(8):1903. doi: 10.3390/biomedicines10081903 (PMC9405850; doi:10.3390/biomedicines10081903)
Supplement: Supplementary file 1 [file biomedicines-10-01903-s001.zip › Supplemetary Figures 1-4.pdf]

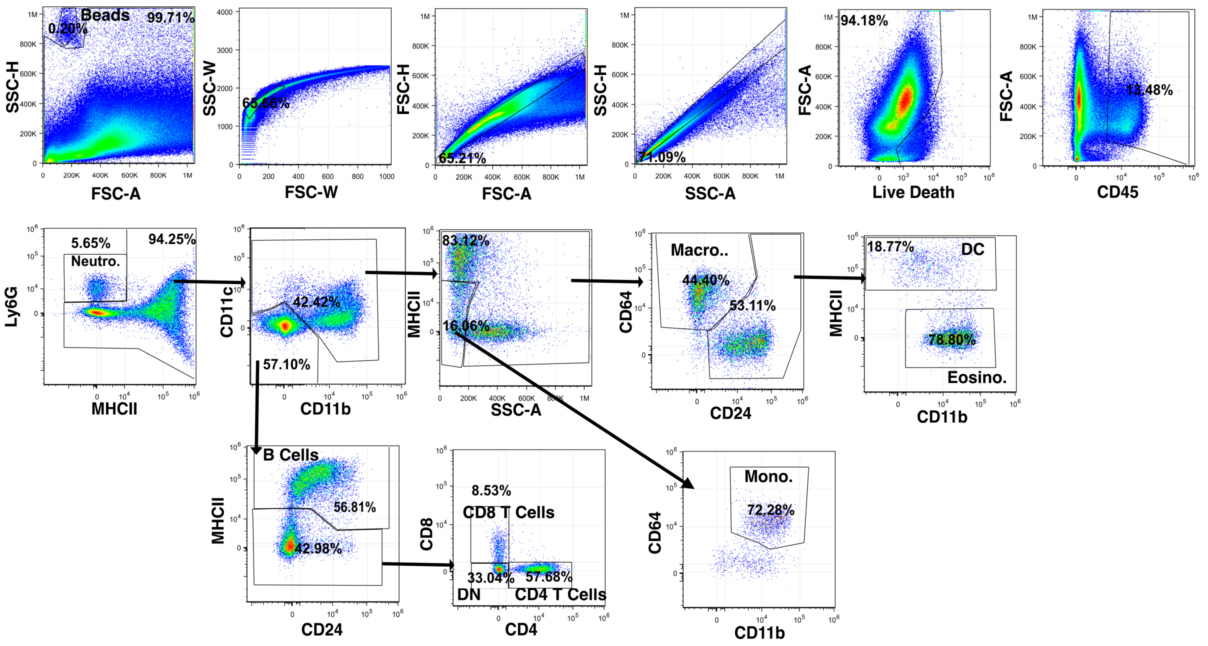

Figure S1: Gating strategy used to identify immune cell subsets in the colon. Gating for colon leukocytes was performed according to a research paper published in 2016 by Yu *et al.* with few modifications[22]. Counting beads were excluded by SSC-H/FSC-A gating. Cell debris and doublets were eliminated by sequential gating on FSC/SSC parameters (SSC-W vs FSC-W, FSC-H vs FSC-A, SSC-H vs SSC-A). Dead cells were excluded and viable leukocytes were identified by CD45 staining. Neutrophils were identified by Ly6G expression. CD11b and CD11c staining was used to differentiate the myeloid cells from CD11b$^-$CD11c$^-$ lymphoid immune cells. B and T lymphocyte subsets were determined by B cell-specific surface markers (MHCII and CD24). Next, CD4$^+$ and CD8$^+$ T cells were determined by gating on CD4 and CD8 cell surface markers. Lymphoid cells negative for CD4 and CD8 lineage markers were considered as double negative (DN) lymphoid cells. MHCII expression and SSC characteristics were used to further subdivide myeloid cell populations. MHCII$^-$ and SSC low populations include monocytes (CD11b$^+$CD64$^+$). Macrophages (CD64$^+$CD24$^-$) were separated from MHCII and SSC high populations. Finally, eosinophils (MHCII$^-$CD11b$^+$) were separated from dendritic cells.

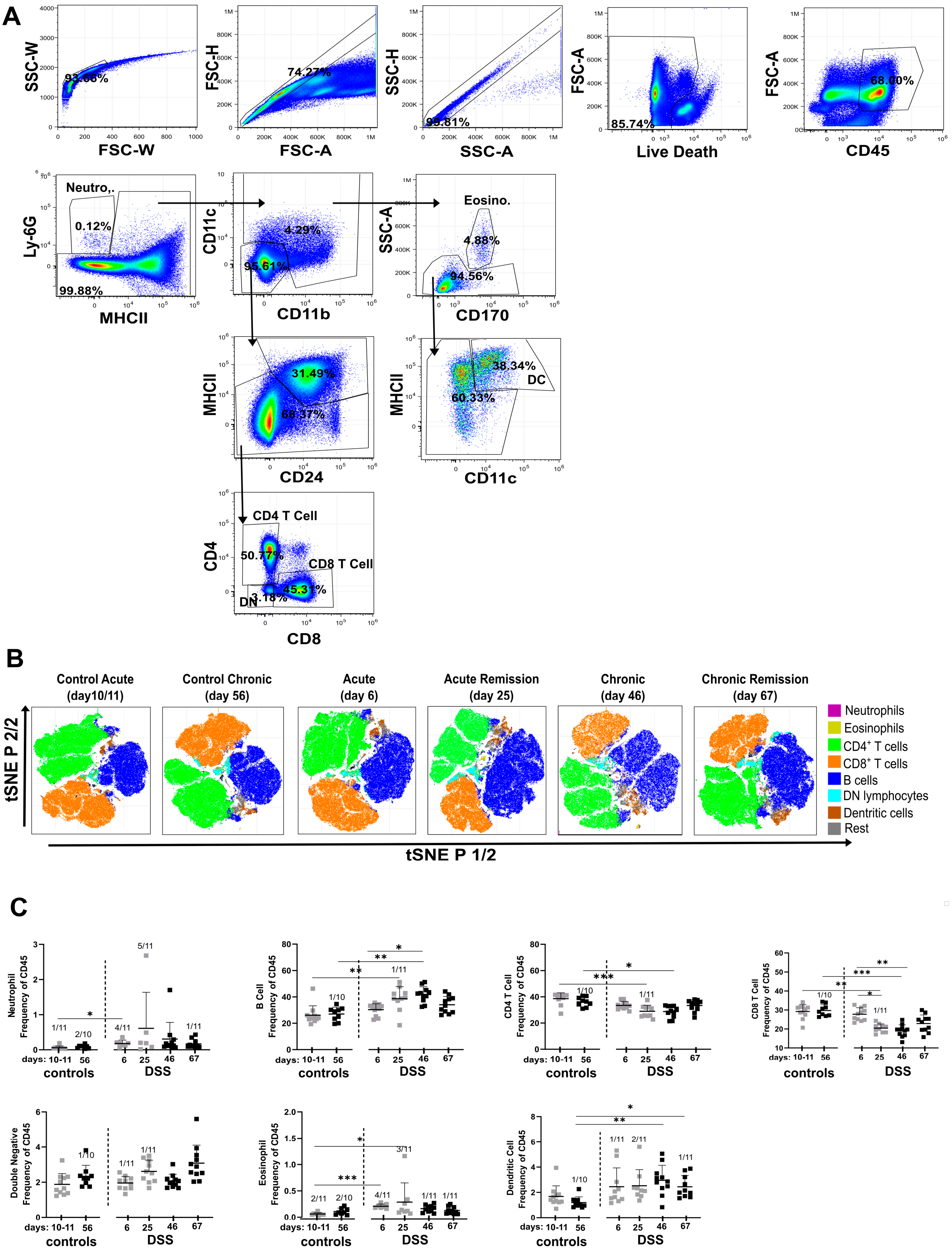

Figure S2: Characterization of immune subsets in mesenteric lymph nodes during consecutive stages of colitis. DSS colitis and respective remission stages were induced as indicated in the main Fig. 1A. At indicated time points, mice were sacrificed, and mesenteric lymph node (MLN) cells were isolated followed by antibody staining and FACS analysis. (A) Gating strategy used to identify the indicated cellular subsets. Cell debris and doublets were eliminated by sequential gating on FSC/SSC parameters (SSC-W vs FSC-W, FSC-H vs FSC-

A, SSC-H vs SSC-A). Dead cells were excluded, and live leukocytes were determined by CD45 staining. Neutrophils were identified by Ly6G marker. CD11b and CD11c staining was used to differentiate the remaining myeloid cells from $CD11b^{-}CD11c^{-}$ lymphoid immune cells. B and T lymphocytes subsets were determined by B cell-specific surface markers (MHCII and CD24). Next, $CD4^{+}$ and $CD8^{+}$ T cells were further determined by using CD4 and CD8 cell surface markers. Lymphoid cells negative for CD4 and CD8 lineage markers were considered as double negative (DN) lymphoid cells. Eosinophils were distinguished by CD170 marker expression within CD11b and CD11c positive myeloid cells. DCs were identified by $MHCII^{+}$ and $CD11c^{+}$ staining within the $CD170^{-}$ cell population. (B) Representative t-SNE plots. Unsupervised clustering of immune cell subsets was performed by *t*-distributed stochastic neighbor embedding (*t*-SNE) and clusters were subsequently identified and color-coded based on manual gating. (C) Frequency of indicated immune cell subsets within the $CD45^{+}$ leukocyte pool. Data were obtained from 2 independent experiments (n = 10 - 11 mice). Grey squares represent control acute (day 10 - 11), acute (day 6) and acute remission (day 25) conditions and black squares represent control chronic (day 56), chronic (day 46) and chronic remission (day 67) groups. Data represent mean with error bars indicating standard deviation. Number of excluded samples is indicated on top of each condition (number of excluded sample/total sample number). Significance was calculated by Kruskal-Wallis test. All groups were compared with each other, significance is indicated only for relevant groups. *** $p < 0.001$, ** $p < 0.01$, * $p < 0.05$.

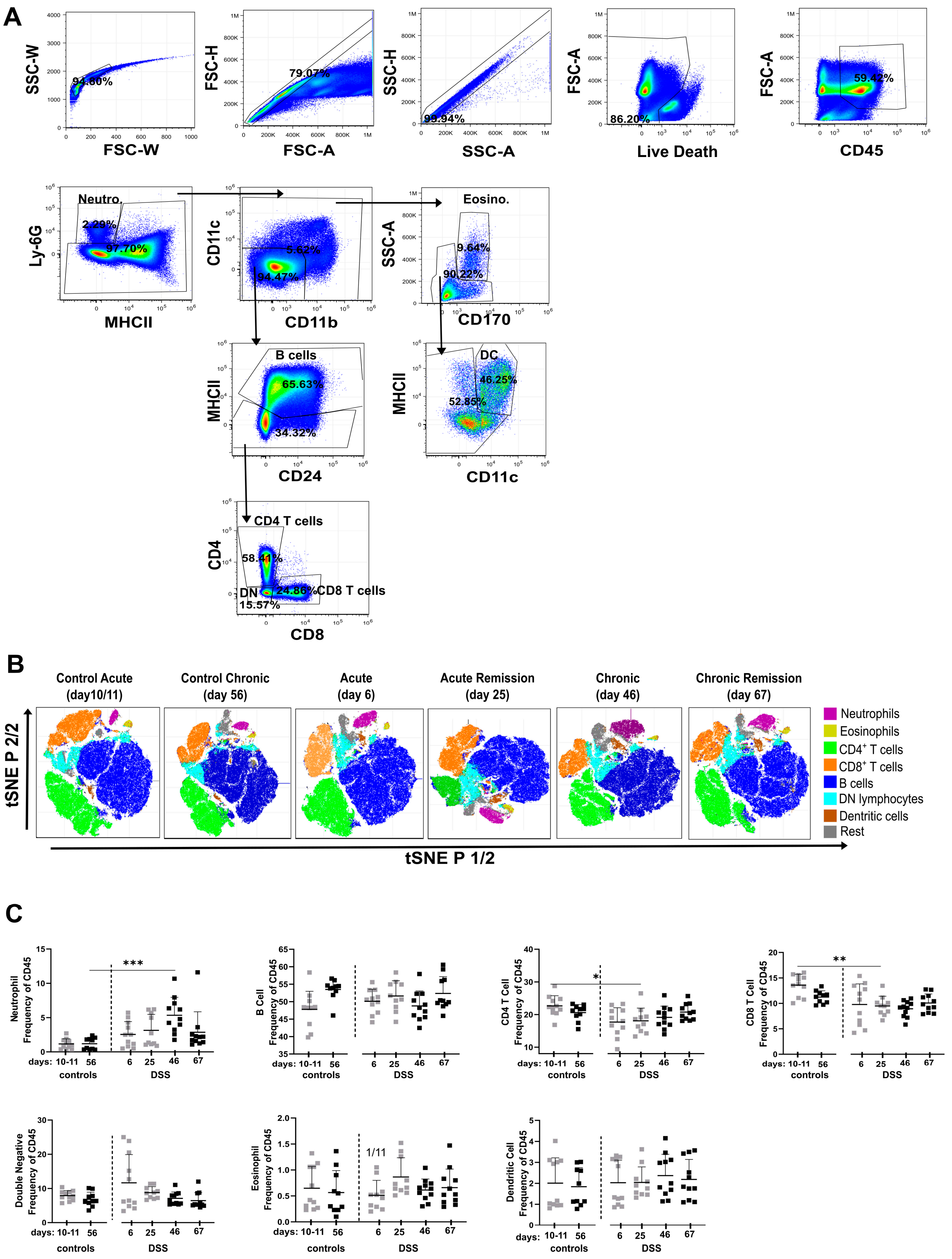

Figure S3: Characterization of immune subsets in the spleen during consecutive stages of colitis. DSS colitis and respective remission stages were induced as indicated in the main Fig. 1A. At indicated time points, mice were sacrificed, and splenocytes were isolated followed by antibody staining and FACS analysis. (A) Gating strategy used to identify the indicated cellular subsets. Cell debris and doublets were eliminated by sequential gating on FSC/SSC parameters (SSC-W vs FSC-W, FSC-H vs FSC-A, SSC-H vs SSC-A). Dead cells were

excluded, and live leukocytes were determined by CD45 staining. Neutrophils were identified by Ly6G marker. CD11b and CD11c staining was used to differentiate the remaining myeloid cells from $CD11b^-CD11c^-$ lymphoid immune cells. B and T lymphocytes subsets were determined by B cell-specific surface markers (MHCII and CD24). Next, $CD4^+$ and $CD8^+$ T cells were further determined by using CD4 and CD8 cell surface markers. Lymphoid cells negative for CD4 and CD8 lineage markers were considered as double negative (DN) lymphoid cells. Eosinophils were distinguished by CD170 marker expression within CD11b and CD11c positive myeloid cells. DCs were identified by $MHCII^+$ and $CD11c^+$ staining within the $CD170^-$ cell population. (B) Representative t-SNE plots. Unsupervised clustering of immune cell subsets was performed by *t*-distributed stochastic neighbor embedding (*t*-SNE) and clusters were subsequently identified and color-coded based on manual gating. (C) Frequency of indicated immune cell subsets within the $CD45^+$ leukocyte pool. Data were obtained from 2 independent experiments (n = 10 - 11 mice). Grey squares represent control acute (day 10 - 11), acute (day 6) and acute remission (day 25) conditions and black squares represent control chronic (day 56), chronic (day 46) and chronic remission (day 67) groups. Data represent mean with error bars indicating standard deviation. Number of excluded samples is indicated on top of each condition (number of excluded sample/total sample number). Significance was calculated by Kruskal-Wallis test. All groups were compared with each other, significance is indicated only for relevant groups. *** $p < 0.001$, ** $p < 0.01$, * $p < 0.05$.

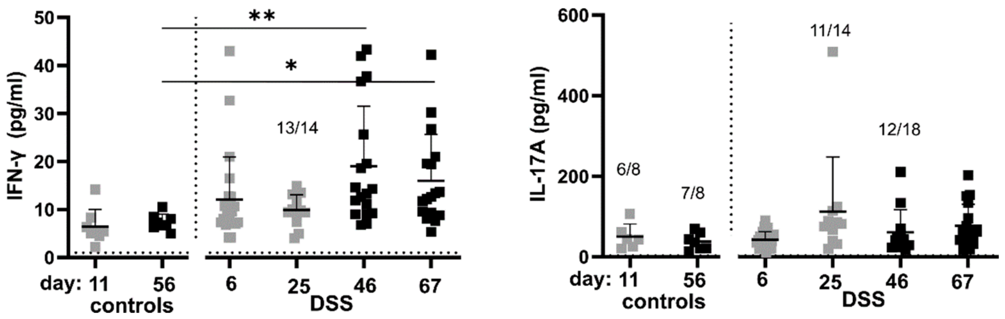

Figure S4: Quantification of IFN-γ and IL-17a in blood plasma during consecutive stages of colitis. DSS colitis and respective remission stages were induced as indicated in the main Fig. 1A. At indicated time points, blood samples were collected, and cytokine levels were determined in plasma. Depicted data matched the following criteria: concentration of the cytokines was above the limit of quantification (LOQ) in at least 71 samples out of 89 samples from all conditions (80 %). Samples with final concentrations that were under the limit of detection (LOD) in at least one out of two technical replicates were excluded from data. Detectable sample numbers out of total samples are indicated in the figure. LOD is indicated by the horizontal dotted line. Data are from n = 8 - 24 mice per group. Grey squares represent control acute (day 11), acute (day 6) and acute remission (day 25) conditions and black squares represent control chronic (day 56), chronic (day 46) and chronic remission (day 67) groups. Data represent mean with error bars indicating standard deviation. Significance was calculated by Kruskal-Wallis test. All groups were compared with each other, significance is indicated only for relevant groups. ** $p < 0.01$, * $p < 0.05$.
